# Supplementary material for: Early childhood caries and its associations with sugar consumption, overweight and exclusive breastfeeding in low, middle and high-income countries: an ecological study
Source: PeerJ. 2020 Oct 1;8:e9413. doi: 10.7717/peerj.9413 (PMC7533058; doi:10.7717/peerj.9413)

Appendix A

Demonstrations of the diagnostics for linearity of relationship

Figure 1: For sugar consumption (with the UAE data in both cases but changing the scale to focus on the bulk of data)

| 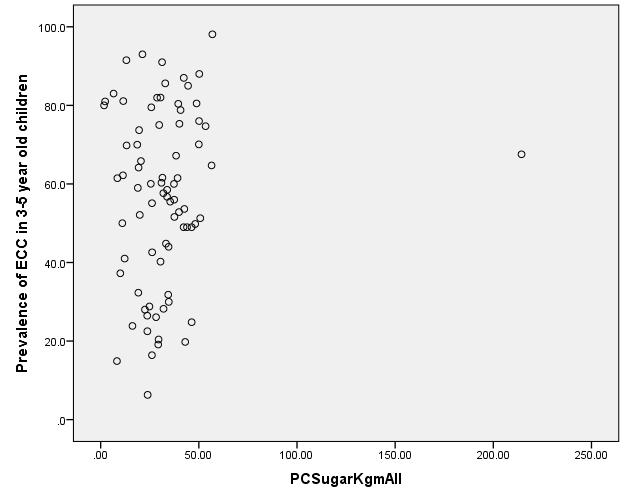 | 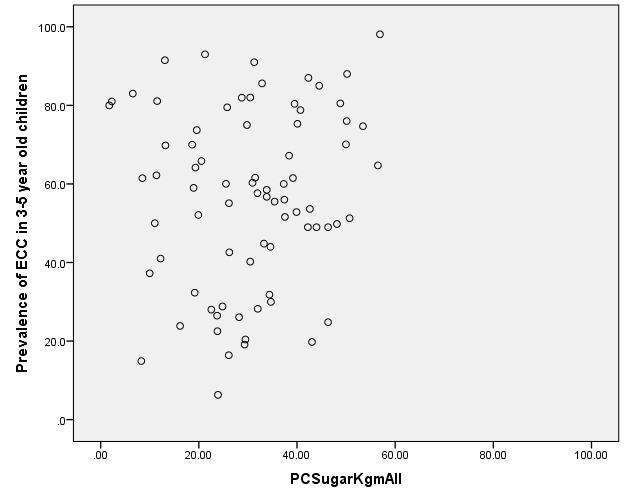 |
| --- | --- |


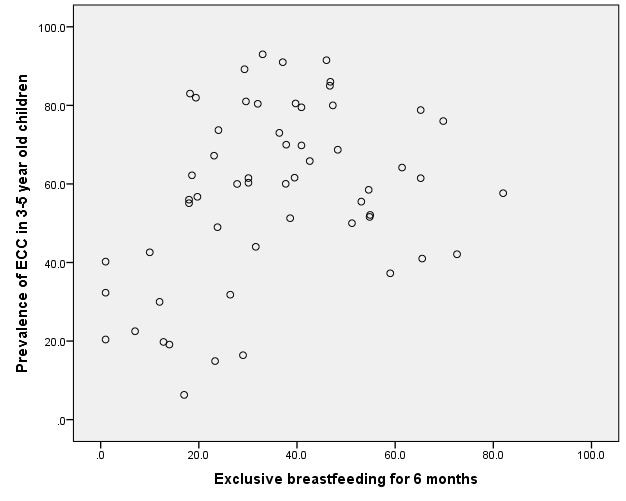


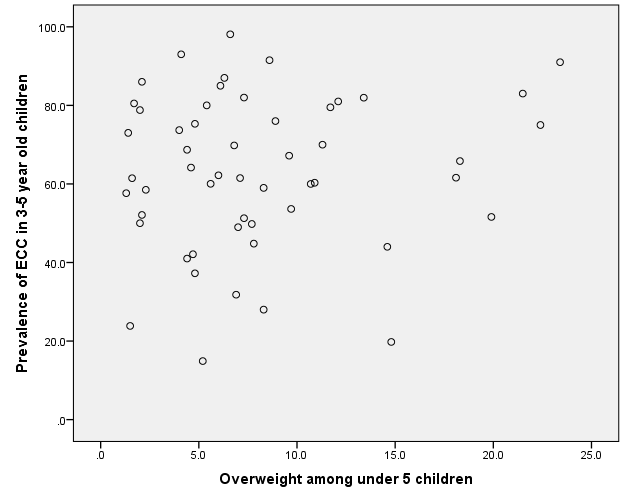


Figure 1: Normal distribution of residuals in the models for sugar consumption

| 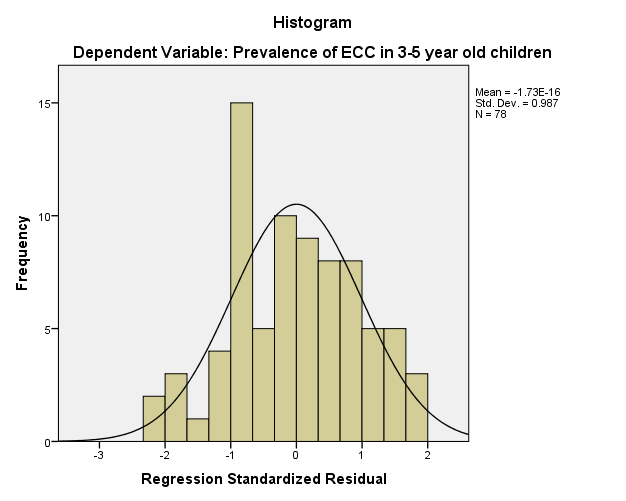 | 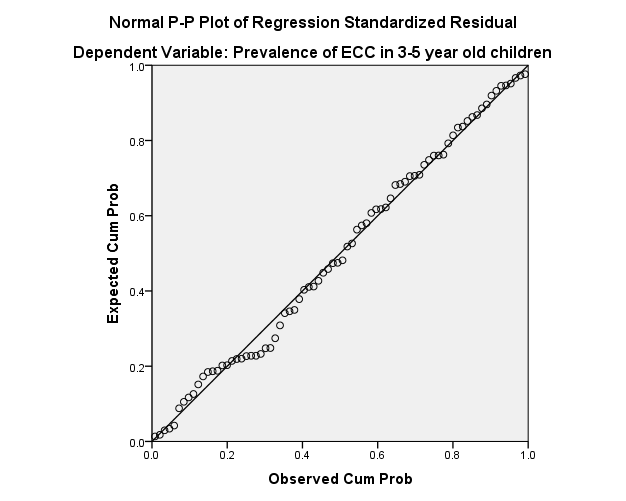 |
| --- | --- |

Figure 3: Normal distribution of residuals in the models for exclusive breastfeeding

| 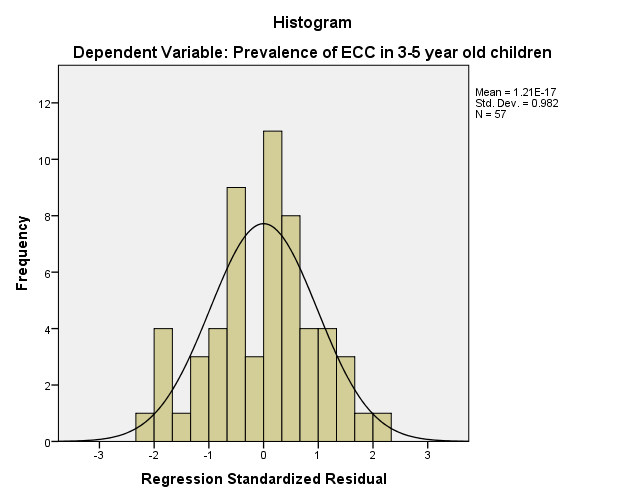 | 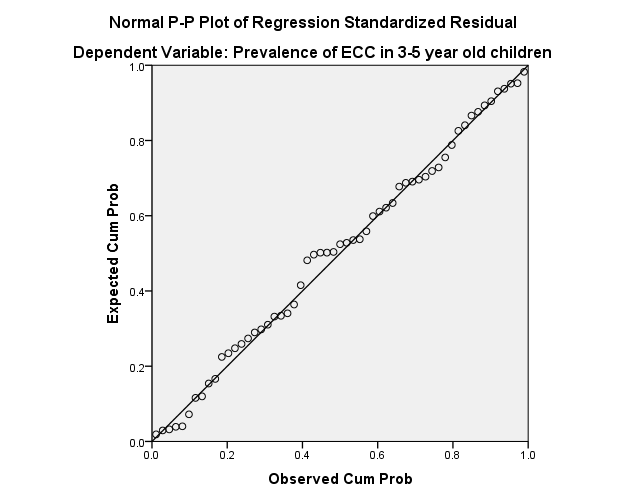 |
| --- | --- |

Figure 4: Normal distribution of residuals in the models for overweight

| 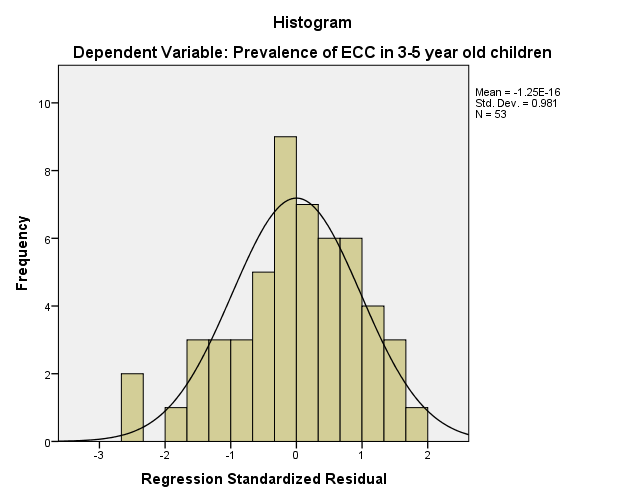 | 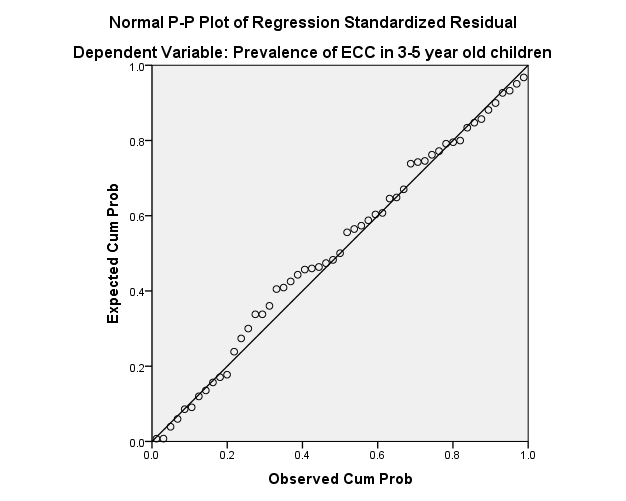 |
| --- | --- |

Figure 5: Constant variance of residuals for per capita sugar consumption


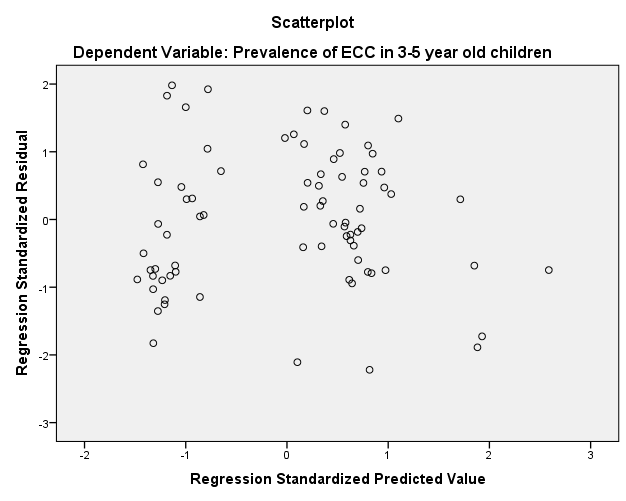


Figure 6: Constant variance of residuals for exclusive breastfeeding


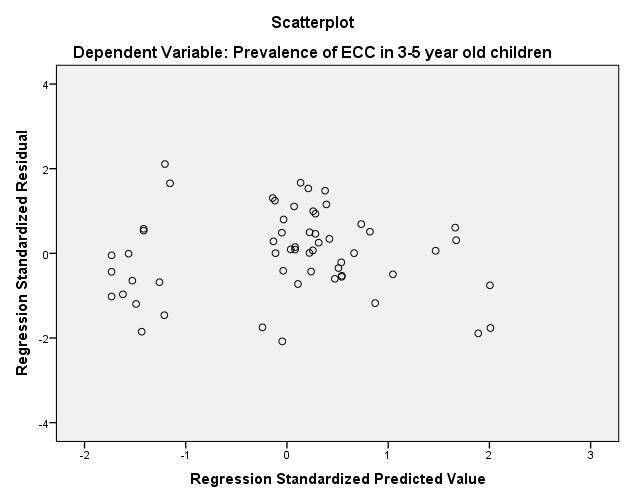


Figure 7: Constant variance of residuals for overweight


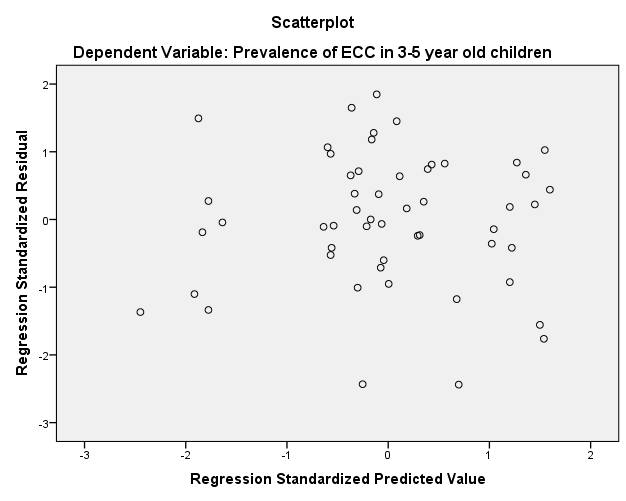

Supplement: Supplemental Information 1 — Classifications of all World Bank member countries (189), and all other economies with populations of more than 30,000. For operational and analytical purposes, economies are divided among income groups according to 2015 gross national income (GNI) per capita, calculated using the World Bank Atlas method. The groups are: low income, $1,025 or less; lower middle income, $1,026–4,035; upper middle income, $4,036–12,475; and high income, $12,476 or more. The effective IDA eligibility threshold is $1,185 or less. Geographic classifications in this table cover all income levels. DA countries are those that lack the financial ability to borrow from IBRD. IDA credits are deeply concessional—interest-free loans and grants for programs aimed at boosting economic growth and improving living conditions. IBRD loans are noncessional. Blend countries are eligible for IDA credits because of their low per capita incomes but are also eligible for IBRD because they are financially creditworthy. Note: The term country, used interchangeably with economy, does not imply political independence but refers to any territory for which authorities report separate social or economic statistics. Income classifications set on 1 July 2016 remain in effect until 1 July 2017. Argentina, which was classified as high income in FY16, is temporarily unclassified pending the expected release of revised national accounts statistics. [file peerj-08-9413-s001.docx]
