# Supplementary material for: Early childhood caries and its associations with sugar consumption, overweight and exclusive breastfeeding in low, middle and high-income countries: an ecological study
Source: PeerJ. 2020 Oct 1;8:e9413. doi: 10.7717/peerj.9413 (PMC7533058; doi:10.7717/peerj.9413)
Supplement: Supplemental Information 2 — Combined data for the prevalence of ECC and per capita sugar consumption per income region were available for 77 countries (4 LICs, 43 MICs and 30 HICs), and ECC and percentage of children exclusively breastfed were available for 57 countries (6 LICs, 37 MICs and 14 HICs). Also, ECC and the percentage of 0-5-year-old children with overweight status were available for 53 countries (6 LICs, 40 MICs and 7 HICs), and ECC and income region data were available for 85 countries (6 LICs, 45 MICs and 34 HICs). [file peerj-08-9413-s002.docx]

Appendix B: Dataset of the study countries

| ID | Country | Per capita Sugar in Kgm | Breastfeeding 6 months | Overweight | ECC | Income level |
| --- | --- | --- | --- | --- | --- | --- |
| 1 | Afghanistan | 1.04 | 43.1 | 5.4 | - | 1 |
| 2 | Albania | 31.29 | 37.1 | 23.4 | 91.0 | 2 |
| 3 | Algeria | 42.45 | 25.4 | 12.4 | - | 2 |
| 4 | Andorra | - | - | - | - | 3 |
| 5 | Angola | 16.24 | 37.4 | 3.3 | - | 2 |
| 6 | Antigua & Barbuda | - | - | - | - | 3 |
| 7 | Argentina | 39.51 | 32.0 | - | 80.4 | 3 |
| 8 | Armenia | 4.04 | 44.5 | 15.2 | - | 2 |
| 9 | Australia | 48.13 | - | 7.7 | 49.8 | 3 |
| 10 | Austria | 26.21 | 10.0 | - | 42.6 | 3 |
| 11 | Azerbaijan | 7.58 | 12.1 | 11.7 | - | 2 |
| 12 | Bahamas | - | - | - | - | 3 |
| 13 | Bahrain | 93.03 | - | - | - | 3 |
| 14 | Bangladesh | 17.45 | 55.3 | 1.7 | - | 2 |
| 15 | Barbados | 31.50 | 19.7 | 12.2 | - | 3 |
| 16 | Belarus | 62.47 | 19.0 | - | - | 2 |
| 17 | Belgium | 34.68 | 12.0 | - | 30.0 | 3 |
| 18 | Belize | 105.70 | 33.2 | 7.6 | - | 2 |
| 19 | Benin | 17.27 | 41.4 | 6.6 | - | 1 |
| 20 | Bhutan | - | 51.4 | 6.0 | - | 2 |
| 21 | Bolivia | 36.61 | 58.3 | 9.4 | - | 2 |
| 22 | Bosnia & Herzegovina | 6.55 | 18.2 | 21.5 | 83.0 | 2 |
| 23 | Botswana | 27.79 | 20.3 | 11.2 | - | 2 |
| 24 | Brazil | 50.71 | 38.6 | 7.3 | 51.3 | 2 |
| 25 | Brunei | 18.93 | - | 8.3 | 59.0 | 3 |
| 26 | Bulgaria | 22.78 | - | - | - | 2 |
| 27 | Burkina Faso | 3.11 | 50.1 | 1.5 | - | 1 |
| 28 | Burundi | 2.88 | 82.3 | 2.2 | - | 1 |
| 29 | Cambodia | 40.70 | 65.2 | 2.0 | 78.8 | 2 |
| 30 | Cameroon | 10.21 | 28.0 | 6.6 | - | 2 |
| 31 | Canada | 32.88 | - | - | 85.6 | 3 |
| 32 | Cape Verde | 39.06 | - | - | - | 2 |
| 33 | Central African Republic | 2.58 | 33.0 | 5.2 | - | 1 |
| 34 | Chad | 2.21 | 0.1 | 2.7 | - | 1 |
| 35 | Chile | 42.62 | - | 9.7 | 53.6 | 3 |
| 36 | Colombia | 40.10 | - | 4.8 | 75.3 | 2 |
| 37 | Comoros | 9.92 | 11.4 | 10.9 | - | 1 |
| 38 | Congo | 17.11 | 32.9 | 4.8 | - | 2 |
| 39 | Congo, DRC | 1.75 | 47.3 | 5.4 | 80.0 | 1 |
| 40 | Costa Rica | 50.53 | 32.5 | 8.1 | - | 2 |
| 41 | Cote d'Ivoire | 14.61 | 23.5 | 3.2 | - | 2 |
| 42 | Croatia | - | - | - | - | 3 |
| 43 | Cuba | 67.62 | 32.8 | - | - | 2 |
| 44 | Cyprus | - | - | - | - | 3 |
| 45 | Czech Republic | 26.13 | 18.0 | - | 55.1 | 3 |
| 46 | Denmark | 23.91 | 17.0 | - | 6.3 | 3 |
| 47 | Djibouti | 193.32 | 12.4 | 10.8 | - | 2 |
| 48 | Dominica | - | - | 8.0 | - | 2 |
| 49 | Dominican Republic | 38.35 | 4.6 | - | - | 2 |
| 50 | Ecuador | 33.26 | - | 7.8 | 44.8 | 2 |
| 51 | East Timor | - | 50.2 | 3.7 | - | 2 |
| 52 | Egypt | 31.47 | 39.5 | 18.1 | 61.6 | 2 |
| 53 | El Salvador | 44.52 | 46.7 | 6.1 | 85.0 | 2 |
| 54 | Equatorial Guinea | - | 7.4 | 9.7 | - | 2 |
| 55 | Eritrea | - | 68.7 | 1.9 | - | 1 |
| 56 | Estonia | - | - | - | 42.0 | 3 |
| 57 | Ethiopia | 9.96 | 56.5 | 2.4 | - | 1 |
| 58 | Fiji | 33.90 | - | - | - | 2 |
| 59 | Finland | 29.49 | 1.0 | - | 20.4 | 3 |
| 60 | France | 24.82 | - | - | 28.8 | 3 |
| 61 | Gabon | 18.76 | 5.1 | 7.7 | - | 2 |
| 62 | Gambia | - | 46.8 | 2.1 | 86.0 | 1 |
| 63 | Georgia | 37.54 | 54.8 | 19.9 | 51.6 | 2 |
| 64 | Germany | 32.00 | - | - | 28.2 | 3 |
| 65 | Ghana | 10.13 | 52.1 | 3.7 | - | 2 |
| 66 | Greece | 19.16 | 1.0 | - | 32.3 | 3 |
| 68 | Guatemala | 45.75 | 53.2 | 4.8 | - | 2 |
| 69 | Guinea | 14.22 | 35.2 | 3.6 | - | 1 |
| 70 | Guinea-Bissau | 2.57 | 52.5 | 2.8 | - | 1 |
| 71 | Guyana | 60.42 | 21.1 | 6.0 | - | 2 |
| 72 | Haiti | 18.33 | 39.9 | 3.8 | - | 1 |
| 73 | Honduras | 41.51 | 30.7 | 5.5 | - | 2 |
| 74 | Hungary | 23.87 | 44.0 | - | - | 3 |
| 75 | Iceland | 37.07 | 13.0 | - | - | 3 |
| 76 | India | 19.90 | 54.9 | 2.1 | 52.1 | 2 |
| 77 | Indonesia | 25.77 | 40.9 | 11.7 | 79.5 | 2 |
| 78 | Iran | 35.42 | 53.1 | - | 55.5 | 2 |
| 79 | Iraq | 28.74 | 19.4 | 13.4 | 82.0 | 2 |
| 80 | Ireland | 23.73 | - | - | 26.5 | 3 |
| 81 | Israel | 56.48 | - | - | 64.7 | 3 |
| 82 | Italy | 28.20 | - | - | 26.1 | 3 |
| 83 | Jamaica | 43.97 | 23.8 | 7.0 | 49.0 | 2 |
| 84 | Japan | 16.17 | - | 1.5 | 23.9 | 3 |
| 85 | Jordan | 15.30 | 22.7 | 5.7 | - | 2 |
| 86 | Kazakhstan | 18.63 | 37.8 | 11.3 | 70.0 | 2 |
| 87 | Kenya | 19.32 | 61.4 | 4.6 | 64.2 | 2 |
| 88 | Kiribati | - | 69.0 | - | - | 2 |
| 89 | South Korea | 30.48 | - | 7.3 | 82.0 | 3 |
| 90 | North Korea | 0.12 | 68.9 | 0.0 | - | 1 |
| 92 | Kuwait | 22.53 | - | 8.3 | 28.0 | 3 |
| 93 | Kyrgyzstan | 13.19 | 40.9 | 6.8 | 69.8 | 2 |
| 94 | Laos | 48.83 | 39.7 | 1.7 | 80.5 | 2 |
| 95 | Latvia | - | 16.0 | - | - | 3 |
| 96 | Lebanon | 53.44 | - | - | 74.7 | 2 |
| 97 | Lesotho | - | 66.9 | 7.4 | - | 2 |
| 98 | Liberia | 6.53 | 54.6 | 3.7 | - | 1 |
| 99 | Libya | 29.81 | - | 22.4 | 75.0 | 2 |
| 101 | Lithuania | - | - | - | 71.8 | 3 |
| 102 | Luxembourg | - | 6.0 | - | - | 3 |
| 103 | Macedonia | 23.15 | 23.0 | 12.4 | - | 2 |
| 104 | Madagascar | 7.31 | 41.9 | - | - | 1 |
| 105 | Malawi | 9.79 | 59.4 | 5.9 | - | 1 |
| 106 | Malaysia | 56.90 | - | 6.6 | 98.1 | 2 |
| 107 | Maldives | 5.81 | 45.3 | 6.5 | - | 2 |
| 108 | Mali | 8.09 | 37.3 | 1.5 | - | 1 |
| 109 | Malta | - | - | - | - | 3 |
| 110 | Marshall Is. | - | 27.3 | - | - | 2 |
| 111 | Mauritania | 86.69 | 41.4 | 1.8 | - | 2 |
| 112 | Mauritius | 31.62 | - | - | - | 2 |
| 113 | Mexico | 39.17 | 30.1 | 7.1 | 61.5 | 2 |
| 114 | Micronesia | - | - | - | - | 2 |
| 115 | Moldova | 35.20 | 36.4 | 4.9 | - | 2 |
| 117 | Mongolia | 13.08 | 46.0 | 8.6 | 91.5 | 2 |
| 118 | Montenegro | - | 16.8 | 22.3 | - | 2 |
| 119 | Morocco | 37.30 | 27.8 | 10.7 | 60.0 | 2 |
| 120 | Mozambique | 10.22 | 41.0 | 5.8 | - | 1 |
| 121 | Myanmar | 11.04 | 51.2 | 2.0 | 50.0 | 2 |
| 122 | Namibia | - | 48.3 | 4.4 | 68.7 | 2 |
| 123 | Nauru | - | 67.2 | 2.8 | - | 3 |
| 124 | Nepal | 8.50 | 65.2 | 1.6 | 61.5 | 1 |
| 125 | Netherlands | 37.41 | 18.0 | - | 56.0 | 3 |
| 126 | New Zealand | 50.20 | - | - | 88.0 | 3 |
| 127 | Nicaragua | 39.65 | 31.7 | 7.3 | - | 2 |
| 128 | Niger | 1.16 | 23.3 | 3.0 | - | 1 |
| 129 | Nigeria | 8.27 | 23.3 | 5.2 | 14.9 | 2 |
| 130 | Norway | 23.79 | 7.0 | - | 22.5 | 3 |
| 131 | Oman | 9.91 | 32.8 | 3.1 | - | 3 |
| 132 | Pakistan | 25.53 | 37.7 | 5.6 | 60.0 | 2 |
| 133 | Palau | - | - | - | - | 2 |
| 135 | Panama | 35.63 | 21.5 | - | - | 2 |
| 136 | Papua New Guinea | 7.36 | 56.1 | 13.8 | - | 2 |
| 137 | Paraguay | 2.27 | 29.6 | 12.1 | 81.0 | 2 |
| 138 | Peru | 50.12 | 69.8 | 8.9 | 76.0 | 2 |
| 139 | Philippines | 21.26 | 33.0 | 4.1 | 93.0 | 2 |
| 140 | Poland | 39.90 | - | - | 52.9 | 3 |
| 141 | Portugal | 18.91 | - | - | - | 3 |
| 142 | Qatar | - | 29.3 | - | 89.2 | 3 |
| 143 | Romania | 11.50 | - | - | 81.1 | 2 |
| 144 | Russia | 42.21 | - | - | 49.0 | 2 |
| 145 | Rwanda | - | 86.9 | 7.4 | - | 1 |
| 152 | Saudi Arabia | 49.98 | - | - | 70.1 | 3 |
| 153 | Senegal | - | 36.4 | 1.4 | 73.0 | 1 |
| 154 | Serbia | 43.04 | 12.8 | 14.8 | 19.8 | 2 |
| 155 | Seychelles | - | - | 10.2 | - | 3 |
| 156 | Sierra Leone | - | 31.4 | 9.8 | - | 1 |
| 157 | Singapore | 46.33 | - | - | 49.0 | 3 |
| 158 | Slovakia | 22.70 | 49.0 | - | - | 3 |
| 159 | Slovenia | - | - | - | - | 3 |
| 160 | Solomon Is. | - | 76.2 | 3.2 | - | 2 |
| 161 | Somalia | 43.41 | 5.3 | 3.0 | - | 1 |
| 162 | South Africa | 34.59 | 31.6 | 14.6 | 44.0 | 2 |
| 163 | Spain | 26.10 | 29.0 | - | 16.4 | 3 |
| 164 | Sri Lanka | 31.94 | 82.0 | 1.3 | 57.7 | 2 |
| 165 | North Sudan | 33.84 | 54.6 | 2.3 | 58.5 | 2 |
| 167 | Suriname | - | 2.8 | 4.0 | - | 2 |
| 169 | Sweden | 29.31 | 14.0 | - | 19.1 | 3 |
| 170 | Switzerland | 46.33 | - | - | 24.8 | 3 |
| 171 | Syria | 20.53 | 42.6 | 18.3 | 65.8 | 2 |
| 173 | Tajikistan | - | 35.8 | 5.4 | - | 2 |
| 174 | Tanzania | 9.99 | 59.0 | 4.8 | 37.3 | 1 |
| 175 | Thailand | 38.38 | 23.1 | 9.6 | 67.2 | 2 |
| 179 | Tunisia | 36.08 | 8.5 | 11.6 | - | 2 |
| 180 | Turkey | 30.94 | 30.1 | 10.9 | 60.3 | 2 |
| 181 | Turkmenistan | - | 58.3 | 5.2 | - | 2 |
| 182 | Tuvalu | - | 34.7 | 6.3 | - | 2 |
| 183 | Uganda | 12.21 | 65.5 | 4.4 | 41.0 | 1 |
| 184 | Ukraine | 33.82 | 19.7 | - | 56.7 | 2 |
| 185 | United Arab Emirates | - | - | - | 67.6 | 3 |
| 186 | United Kingdom | 30.47 | 1.0 | - | 40.2 | 3 |
| 187 | United States | 34.39 | 26.4 | 6.9 | 31.8 | 3 |
| 188 | Uruguay | 42.94 | - | 7.6 | - | 3 |
| 189 | Uzbekistan | - | 23.8 | 12.8 | - | 2 |
| 190 | Vanuatu | - | 72.6 | 4.7 | 42.1 | 2 |
| 192 | Venezuela | 42.31 | - | 6.3 | 87.0 | 2 |
| 193 | Vietnam | 19.57 | 24.0 | 4.0 | 73.7 | 2 |
| 194 | Yemen | 25.49 | 9.7 | 1.8 | - | 2 |
| 195 | Zambia | 21.64 | 72.0 | 7.3 | - | 2 |
| 196 | Zimbabwe | 23.59 | 47.1 | 4.6 | - | 1 |
| 345 | China | 11.36 | 18.6 | 6.0 | 62.2 | 2 |

Income level: 1= LIC, 2= MIC, 3= HIC
