# Supplementary material for: Early childhood caries and its associations with sugar consumption, overweight and exclusive breastfeeding in low, middle and high-income countries: an ecological study
Source: PeerJ. 2020 Oct 1;8:e9413. doi: 10.7717/peerj.9413 (PMC7533058; doi:10.7717/peerj.9413)

Appendix C

Regression diagnostics

Linearity of relationship

Figure 1: For sugar consumption (with the UAE data in both cases and changing the scale to focus on the bulk of data in the figure on the right)

| 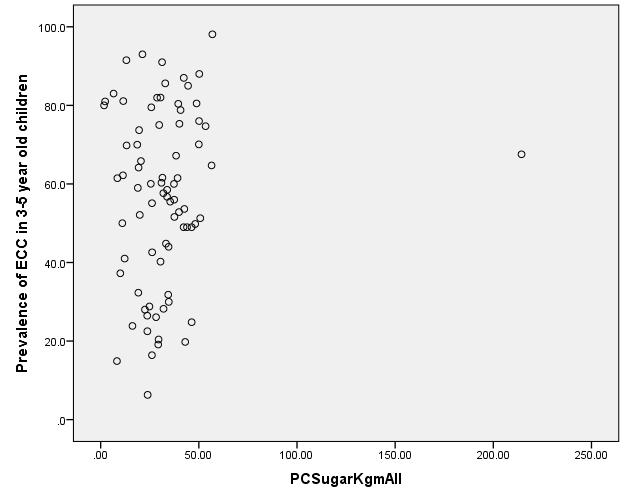 | 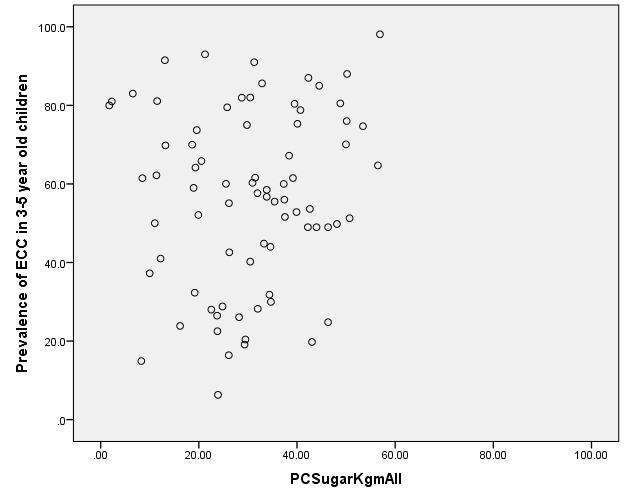 |
| --- | --- |


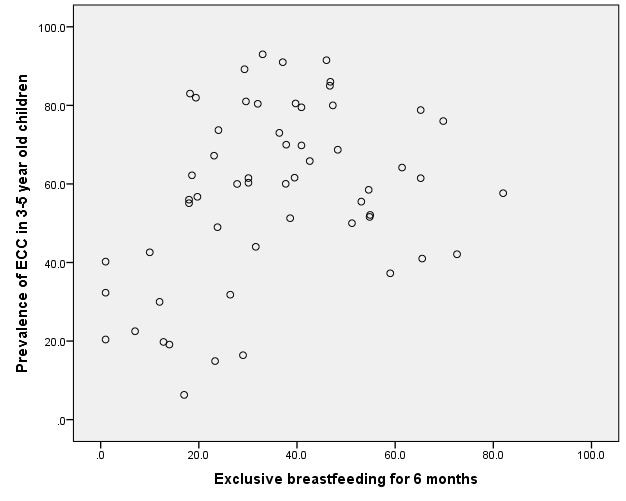


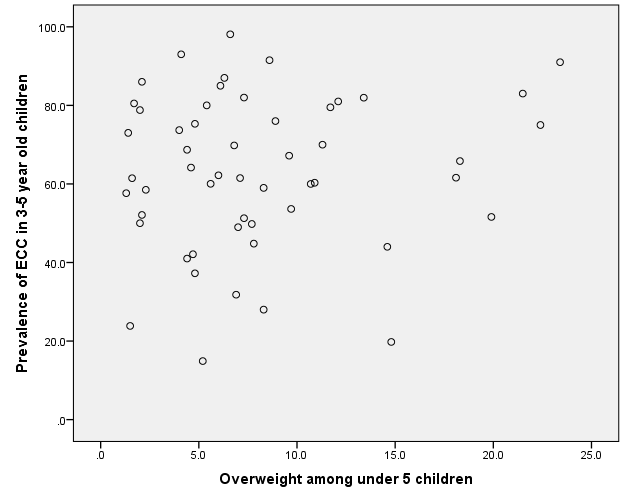


Figure 1: Normal distribution of residuals in the models for sugar consumption

| 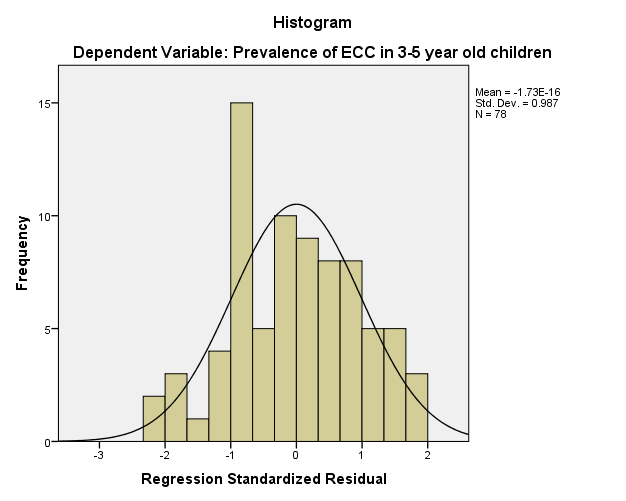 | 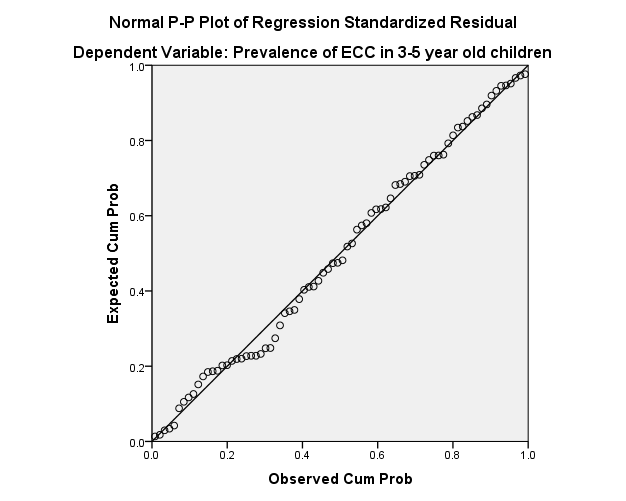 |
| --- | --- |

Figure 3: Normal distribution of residuals in the models for exclusive breastfeeding

| 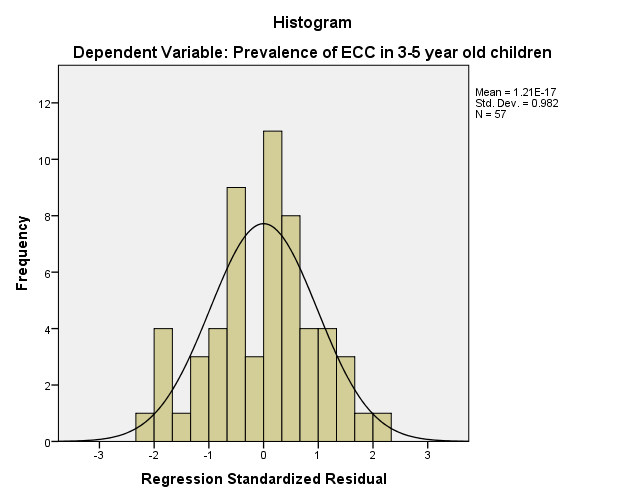 | 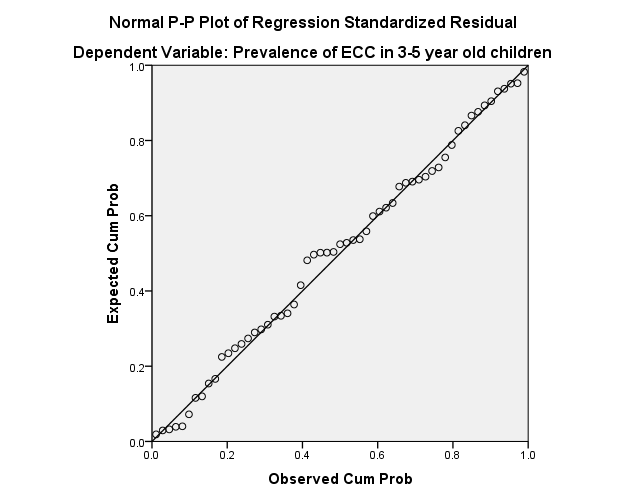 |
| --- | --- |

Figure 4: Normal distribution of residuals in the models for overweight

| 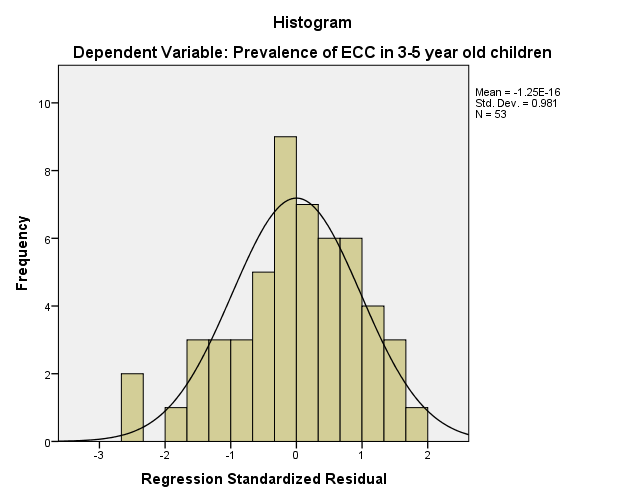 | 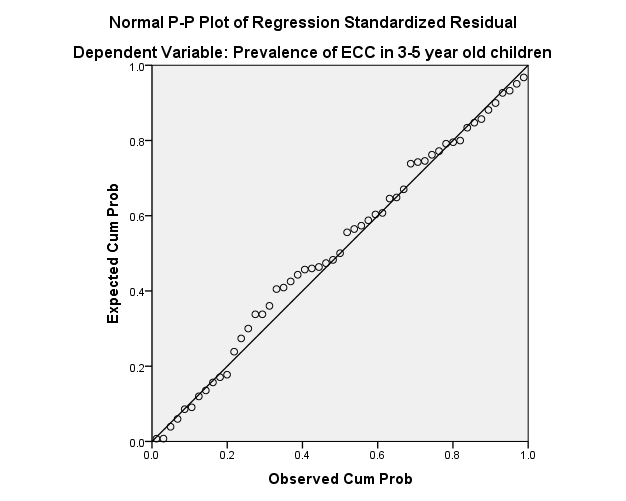 |
| --- | --- |

Figure 5: Constant variance of residuals for per capita sugar consumption


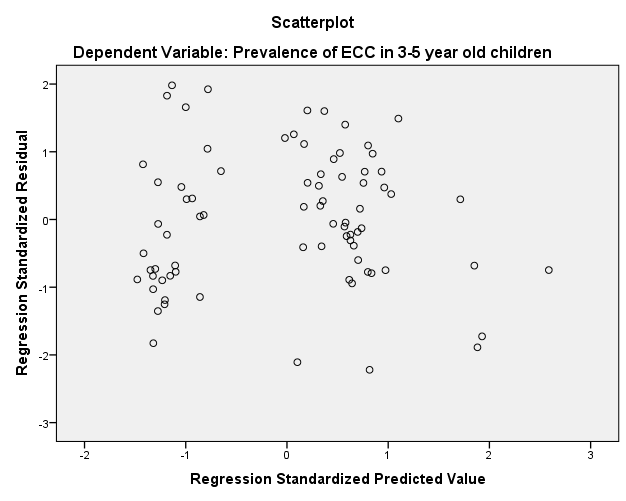


Figure 6: Constant variance of residuals for exclusive breastfeeding


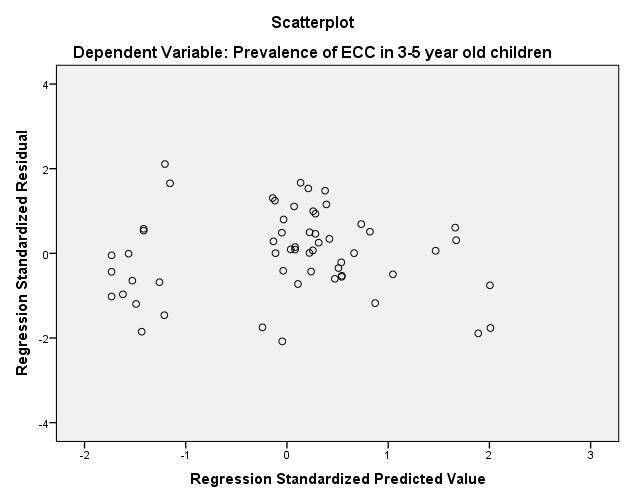


Figure 7: Constant variance of residuals for overweight


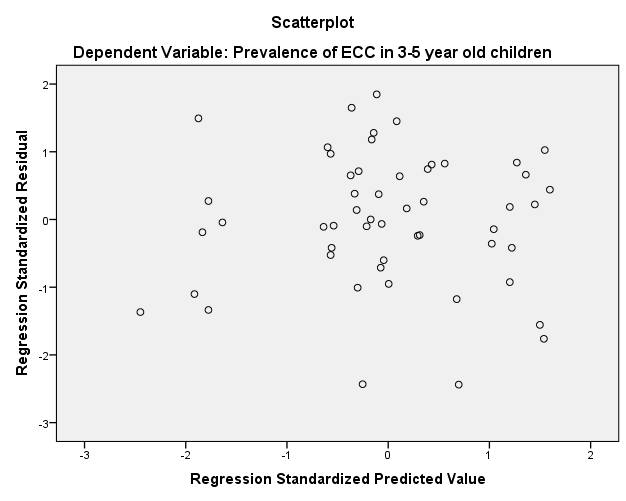

Supplement: Supplemental Information 3 [file peerj-08-9413-s003.docx]
